# Different Threshold of Malignancy for *RAS*-like Thyroid Tumors Causes Significant Differences in Thyroid Nodule Practice

**DOI:** 10.3390/cancers14030812

**Published:** 2022-02-05

**Authors:** Kennichi Kakudo

**Affiliations:** Department of Pathology, Cancer Genome Center and Thyroid Disease Center, Izumi City General Hospital, Izumi 594-0073, Japan; kakudo@thyroid.jp; Tel.: +81-725-41-1331

**Keywords:** papillary thyroid carcinoma, *BRAFV600E*, *RAS*, follicular adenoma, noninvasive follicular thyroid neoplasm with papillary-like nuclear feature, borderline tumor, uncertain malignant potential

## Abstract

**Simple Summary:**

Histopathological diagnosis is prone to significant observer variation in the diagnosis of papillary thyroid carcinomas (PTCs) due to different thresholds of malignancy for *RAS*-like nuclear features (worrisome nuclear features of PTC). *RAS*-like PTCs in Western practice are differently classified by most Asian pathologists into follicular adenomas when they are not invasive, follicular carcinomas when they are invasive, and follicular variant PTCs when they have fully developed nuclear features. This commentary emphasizes that this observer variation in diagnosing *RAS*-like thyroid tumors among practices underlies several issues in thyroid nodule practice.

**Abstract:**

Histopathological diagnosis of papillary thyroid carcinomas (PTCs) is prone to significant observer variation due to different thresholds of *RAS*-like nuclear changes among pathologists. This gap recently widened due to a defensive attitude by Western pathologists where malpractice litigation is significant. Cases with delicate *RAS*-like nuclear changes are follicular adenomas when they are noninvasive, follicular carcinomas when invasive, and follicular variant PTCs when they have fully developed PTC-type nuclear features in Asian practice. The different diagnostic threshold of PTC nuclear features resulted in a high (50–90%) incidence of *BRAFV600E* mutation of PTCs in most Asian countries, whereas it was low (35–50%) in most Western patient cohorts. The contamination of indolent *RAS*-like tumors in the malignant PTC category in Western patient cohorts explains why the *BRAFV600E* gene test identifies aggressive PTCs. However, the *BRAFV600E* test has no prognostic value for Asian PTC patients because most biologically benign or low-risk *RAS*-like tumors are excluded from PTC. All prognostic analyses of thyroid carcinomas before 2017 must be re-evaluated because most clinical guidelines were established based on data obtained from Western patient cohorts where a significant number of indolent *RAS*-like tumors were misclassified in the malignant category.

## 1. Introduction

**Figure 1:**
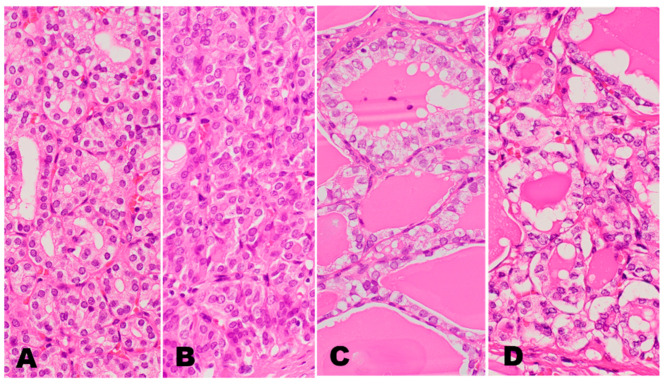
Nuclear features of encapsulated follicular pattern tumors. (**A**): nuclear score 0 in a follicular adenoma, (**B**): nuclear score 2 in a NIFTP (nuclear enlargement 1, membrane irregularity 1 and chromatin clearing 0), (**C**): nuclear score 3 (nuclear enlargement 1, membrane irregularity 1, chromatin clearing 1) in a NIFTP defined by Nikiforov et al. [22]. (**D**): fully developed nuclear features of a *BRAF*-like encapsulated follicular subtype PTC. (HE stain, ×40).

There are several issues in thyroid nodule practice internationally. The author hypothesizes significant misunderstandings and poor mutual incomprehension among thyroid researchers to be the major causes [1]. The author focuses on two debates and possible explanations in this commentary: (1) why the prevalence of *BRAFV600E* mutation was high (50–90%) in Asian papillary thyroid carcinoma (PTC) patient cohorts but low (35–50%) in most Western PTC cohorts [2], and (2) why the *BRAFV600E* gene test identifies high-risk PTCs in Western patients [3,4,5,6], but the test results were not reproducible in most Asian patients [7,8,9,10,11,12,13]. Conflicts between different studies stem from a variety of reasons, including patient selection, different histological types included in the analysis, different methods applied for *BRAFV600E* testing, and epidemiological factors [2,6,14,15,16]. The author believes it was due in significant part to an inconsistent diagnostic threshold of malignancy for *RAS*-like follicular pattern neoplasms among pathologists, in addition to the other factors mentioned above. Asian pathologists often classify delicate nuclear features of *RAS*-like follicular pattern thyroid tumors as benign follicular adenomas when non-invasive, malignant tumors (follicular thyroid carcinoma: FTC) when invasive, and follicular variant PTCs when they have fully developed PTC-type nuclear changes [1,17,18]. However, Western pathologists often diagnose follicular pattern tumors with delicate nuclear features as malignant tumors (follicular variant PTC) regardless of invasive growth [4,19,20,21,22] (Figure 1). The benign/malignant observer variation in delicate nuclear features (worrisome for PTC) of non-invasive encapsulated *RAS*-like neoplasms is significant among pathologists, particularly between Asian and Western pathologists [1,17,18,23,24,25]. The guest editor of this Special Issue of molecular advances and targeted therapy in Asian thyroid practice believes this caused significant differences between Asian and Western thyroid nodule practices, which are evident from the above two debates and other studies in this Special Issue.

## 2. *RAS* Mutated Follicular Pattern Thyroid Tumors

A significant number of follicular adenoma (20–40%), noninvasive follicular thyroid neoplasm with papillary-like nuclear features (NIFTP) (30–60%), uncertain malignant potential (UMP) (14–20%), follicular variant PTC (15–43%), FTC (30–50%) and poorly differentiated thyroid carcinoma (18–50%) cases have *RAS* mutations and are *RAS*-mutated follicular pattern thyroid tumors [4,21,22,26,27,28,29,30,31,32,33]. This means that *RAS* gene alterations cannot be evidence of malignancy. Guan et al. and Soares et al. concluded in their excellent papers that the most prevalent molecular alterations in follicular patterned thyroid tumors are *RAS* mutations and they do not carry prognostic significance [15,16]. They suggested that the nuclear morphology often observed in *RAS*-like tumors [*RAS*-like dysplasia (nuclear enlargement, membrane irregularity, and chromatin clearing)] cannot be evidence of malignancy [15,16]. As a result, the presence of invasion and/or metastasis provides distinction between benign and malignant in *RAS*-like follicular pattern tumors [31]. This diagnostic schema for both follicular adenoma/FTC and NIFTP/follicular variant PTC will be incorporated in the forthcoming 5th edition World Health Organization (WHO) classification of thyroid tumors to be published in 2022 [34]. However, Western pathologists have significantly modified the diagnostic criteria for PTC type malignancy in the past 30 years, and the characteristic nuclear morphology of *RAS*-like tumors was accepted as the most important diagnostic criteria for PTC-type malignancy regardless of invasion and metastasis [19,35]. This characteristic nuclear morphology was previously termed PTC-type nuclear features, and it was renamed papillary-like nuclear features by the international NIFTP working group to distinguish it from fully developed nuclear features diagnostic of *BRAF*-like PTC [22]. Cases with such delicate nuclear features alone were accepted as malignancy and treated accordingly, regardless of growth (papillary or follicular) pattern and invasion in past Western practice [19,35]. However, most Asian and some Western pathologists resisted this wave because the overdiagnosis of these indolent encapsulated follicular pattern thyroid tumors inevitably causes significant treatment related complications and places psychological burden on the patient without prognostic benefits [36,37,38]. Liu et al. at the Memorial Sloan-Kettering Cancer Center revealed no recurrence in 43 cases of noninvasive encapsulated follicular variant PTC with a median follow-up of 11.1 years [20]. They emphasized that strong consideration should be given to reclassifying encapsulated follicular variant PTC as an entity that is close to the follicular adenoma/FTC class of tumor [20], and the guest editor Kakudo et al. proposed a borderline tumor category to accept those indolent thyroid tumors in the thyroid tumor classification [36,37,38].

## 3. *RAS*-like PTC

The diagnostic threshold of PTC-type malignancy in Western pathology practice has been significantly modified since the 1980s, and cases with delicate nuclear changes in *RAS*-like tumors were accepted as evidence of malignancy [19,35]. This diagnostic tendency (low threshold of diagnostic nuclear features of PTC) made non-invasive encapsulated follicular variant PTC the most frequent histological subtype in Western patient cohorts [4,19,22,39,40], but not in Asian patients where only fully developed nuclear features indicated follicular variant PTCs [24,25,26,41,42,43,44]. This different threshold for malignancy in follicular pattern tumors was confirmed as the cause of the significant observer variation among pathologists when evaluating the same specimens [17,18,45,46,47,48], particularly between Asian and Western pathologists [17,18,45]. It was attributed to non-scientific factors, particularly severe malpractice climates and defensive clinical attitudes [49,50]. 

Labargo et al. demonstrated that states with a higher malpractice payout rate had a higher thyroid cancer incidence, and the author suggested that the malpractice climate is a social determinant for being diagnosed with thyroid cancer [49]. This trend in North America has accelerated over the last three decades. Renshaw and Gould explained their motivation to over-diagnose uncertain cases rather than under-diagnose them in North American practice [51]. The gap between North America and the rest of the world became wider, and non-invasive encapsulated follicular variant PTC accounted for nearly 30% of PTC in North American practice [22,39,40,52,53], whereas it was low (1–5%) in most Asian thyroid practices [24,25,41,42,43,44,48], some European institutes [14,54,55,56], and a few US centers [25,57]. Hodak et al. discussed this overdiagnosis in their excellent commentary, suggesting that for many patients with thyroid cancer, we may often be violating the important dictum primum non nocere (first, do no harm patients) [58]. Chan et al. recommended stricter criteria for the diagnosis of encapsulated follicular pattern tumors [59].

## 4. PTC in Asian Practice and Western Practice

**Figure 2:**
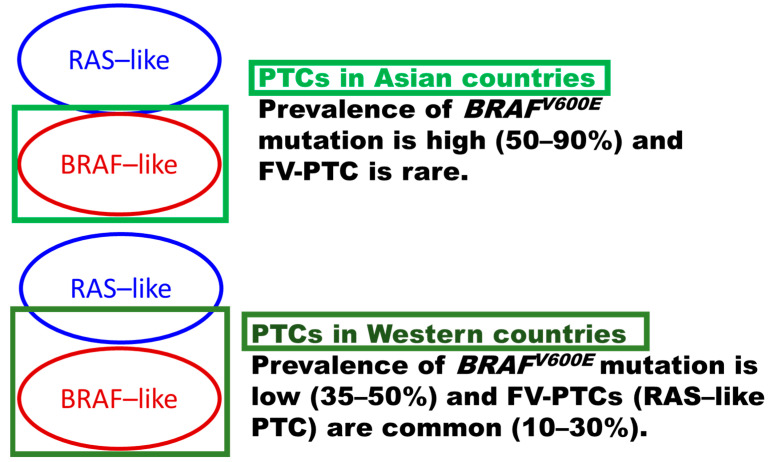
The green square (upper field) indicates the definition of Asian papillary thyroid carcinoma (PTC), which covers all *BRAF*-like PTCs but rare *RAS*-like PTCs. It shows why the prevalence of *BRAFV600E* is high (50–90%), and *RAS*-like follicular variant PTC (FV-PTC) is rare in the Asian definition of PTC. The lower dark green square shows the Western definition of PTC, which covers both *BRAF*-like PTC with fully developed PTC-type nuclear features and *RAS*-like PTC with delicate nuclear features.

As cases with delicate nuclear changes in *RAS*-like tumors were accepted as PTC in Western practice [35] and the 3rd edition WHO classification [19], the proportion of follicular variant PTC with *RAS* mutation was high in Western practice before NIFTP introduction (Figure 2) [2]. On the other hand, *RAS* mutations were rare in Asian PTCs because delicate nuclear features of *RAS*-like tumors were not accepted as evidence of PTC-type malignancy. They were often classified into follicular adenoma when non-invasive, FTC when invasive, and follicular variant PTC when *BRAF*-like fully developed nuclear features were observed in most Asian practices (Figure 1). The author of this commentary emphasizes that the Asian diagnostic threshold of PTC nuclear features was an international standard in past pathology practice, and invasive encapsulated follicular pattern tumors with delicate nuclear changes were FTCs internationally even in the USA, which was confirmed by Cipriani et al. They reviewed 66 cases of FTCs diagnosed between 1965 and 2007 in an academic center in the USA, and reported that 24 (36%) FTCs were reclassified as PTCs under their diagnostic criteria in 2014, which means encapsulated follicular pattern tumors with subtle nuclear changes were historically FTCs (1965–2007), but cases with subtle nuclear features were PTC in 2014 [60]. Mehrzad et al. examined 953 thyroid nodules treated between 2004 and 2013 in another academic center in the USA, and demonstrated that the incidence of follicular adenomas decreased, resulting in a greater than 10-fold increase in the ratio between follicular variant PTC and follicular adenomas during the study period [61]. They suggested that many nodules previously diagnosed as follicular adenoma had been labeled as encapsulated (noninvasive) follicular variant PTC in recent years [61]. Widder et al. examined their 185 patients with follicular neoplasm (their initial diagnoses were 118 benign, 56 follicular variant PTCs, and 11 FTCs) treated between 1993 and 2003 at an academic center in Canada [62]. Their pathologists re-reviewed the 185 follicular neoplasms under the diagnostic criteria in 2007, and 35 were reclassified from a benign diagnosis to a re-reviewed malignant diagnosis, with 5 reclassified as minimally invasive FTC, 4 as occult PTC, and 26 (74%) as follicular variant PTC. Of the 26 follicular variant PTCs, the author of this commentary assumes that most were not high-risk invasive encapsulated follicular variant PTCs but possible NIFTPs, as none of them developed recurrence or persistent disease with a mean follow-up of 105 months [62]. Of note, certain tumors historically considered FTC are now considered PTC, and benign follicular adenomas according to the past criteria are malignant (follicular variant PTC) by the 3rd edition WHO classification. 

**Figure 3:**
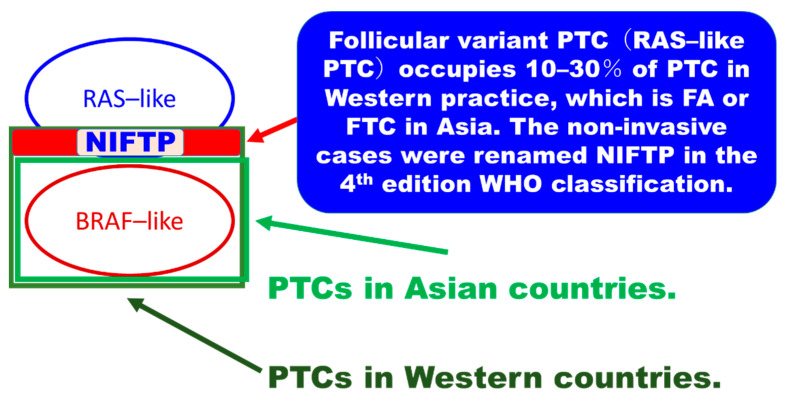
Difference between the Asian and Western definitions of papillary thyroid carcinoma. There was a non-overlapping part (red highlighted) between the Western and Asian definitions of papillary thyroid carcinoma (PTC). When superimposing Western PTCs on Asian PTCs, the non-overlapping part (red part) is the *RAS*-like tumor, which is morphologically a follicular variant PTC that accounts for 10–30% of the US PTC cohort. It was renamed NIFTP when noninvasive and downgraded to the borderline (not malignant) tumor category in the 4th edition WHO classification of thyroid tumors in 2017 [22,31]. NIFTP: noninvasive follicular thyroid neoplasm with papillary-like nuclear features.

These studies confirmed that the delicate nuclear features suggesting PTC observed in *RAS*-like follicular pattern tumors were not PTC in past USA pathology practice. This editor emphasizes that the definition of PTC is different between Asian (upper) and Western (lower) thyroid pathology practice, and the definitions correspond to the diagnostic schema in the 4th and 3rd edition WHO classification, respectively (Figure 2). When superimposing Western PTCs on Asian PTCs, the non-overlapping part (red part in Figure 3) is the *RAS*-like PTC, which is a follicular variant PTC that accounts for 10–30% of US PTC cohorts (Figure 3). Noninvasive follicular variant PTCs were renamed NIFTP (not a malignant tumor) in the 4th edition WHO classification [31] and most invasive follicular variant PTCs were excluded from PTC and classified as FTC in Asian practice (Figure 3).

## 5. Why the Prevalence of *BRAFV600E* Mutation Was High in Asian PTC Cohorts but Low in Most Western PTC Cohorts

When comparing Asian and Western PTC, the different diagnostic criteria explained in the above section significantly impacted the proportion of driver genes. Most studies from Asian countries reported a reasonably high (50–90%) prevalence of *BRAFV600E* mutation, as significant numbers of *RAS*-like tumors were excluded from PTC as follicular adenoma, NIFTP, or FTC (Figure 2). In contrast, the prevalence of *BRAFV600E* mutation in Western PTC patient cohorts is low (35–50%) because a significant number of *RAS*-like follicular pattern tumors (so-called noninvasive follicular variant PTC) contaminated PTCs due to lax criteria for PTC-type nuclear features. In recent two Asian studies using a next-generation sequencing method, the *BRAFV600E* mutation rate was 57% and 65%, respectively [63,64]. Although these values were within the Asian range, they are slightly lower than those of the other Asian studies using the PCR-sequencing method. While in Western studies, the TCGA research network reported a slightly higher (59.7%) [30] *BRAFV600E* mutation in US patients than those (35–50%) of most Western series. Those highly sophisticated studies suggested the real prevalence of *BRAF* mutation in PTCs may be within the above three prevalence (57–65%). It means the high prevalence in earlier Asian studies might suffer from PCR noise and those Western studies with low prevalence might suffer from low sensitivity problems. Therefore, the variable prevalence of *BRAF* mutation in PTCs among countries remains a significant debate until the technical issue is completely solved. However, as shown in the Figure 2 and Figure 3, the author of this commentary suggests the proportion of *RAS*-like PTC decides the prevalence of *BRAFV600E* mutation of all PTCs regardless of the test method and background *BRAF* prevalence. Please note the incidence of infiltrative FV-PTC, encapsulated FV-PTC, and NIFTP in 26604 PTCs in 6 Asian countries reported by Bychkov et al. [24] were 1.4% (infiltrative FV-PTC), 1% (invasive encapsulated FV-PTC), and 0.8% (NIFTP), (all FV-PTC by the 3rd edition WHO classification were 4%), while that prevalence of US patients by Fagin et al. [39] was 6% (infiltrative FV-PTC), 4% (invasive encapsulated FV-PTC) and 17% (NIFTP), (all FV-PTCs by the 3rd edition WHO classification were 27%). The NIFTP (10–20%) alone lowered the *BRAF* prevalence in the Western practice significantly as they were *RAS*-like PTCs in Western practice.

Furthermore, the prevalence of *BRAFV600E* was consistently high in Western countries [4,65,66,67,68], similar to Eastern countries [69], when limited to classic and tall cell variant PTC. (Table 1). This observation supports the author’s conclusion that the low frequency of *BRAF* mutation in Western PTC patients is due to the high-frequency follicular variant PTC of *BRAF* wild-type in Western practice.

## 6. Why the *BRAFV600E* Gene Test Identifies High-Risk PTCs in Western Patients, but It Was Not Reproducible in Most Asian Patient Cohorts

More recently, *BRAFV600E* mutation was suggested as an essential adjunct in predicting adverse prognosis in PTC [3,5]. Xing M et al. reported a significant association between *BRAF* mutation and extrathyroidal invasion, lymph node metastasis, and advanced tumor stage at initial surgery in 219 PTCs [3]. A meta-analysis by Li et al. confirmed that *BRAF* mutation is associated with lymph node metastases, tumor stage, extrathyroidal extension, tumor size, male sex, multifocality, absence of capsule, classic PTC, and tall-cell variant PTC in PTC [6]. Therefore, it was once accepted as a biomarker tailoring postoperative management of PTC patients. However, the analysis by Xing et al. was carried out on a multicenter Western PTC cohort where contamination of biologically indolent follicular variant PTC was significant. Their PTCs included 77 (35%) follicular pattern PTCs and 66 (86%) were *BRAF*-wild type. Thus, the editor assumes that most were indolent *RAS*-like follicular pattern tumors including significant numbers of NIFTP. The comparison of two PTC groups (*BRAFV600E* mutated vs. *BRAF*-wild type) by Xing M et al. was biased due to the substantial contamination of biologically indolent NIFTP in the *BRAF* wild type group; therefore, the editor emphasizes that *BRAFV600E* mutation in PTC in earlier studies must be interpreted with caution.

The editor concludes the *BRAFV600E* mutation alone does not carry prognostic significance in PTCs based on the following [16,70,71]: (1) *BRAFV600E* mutation was found in clinically insignificant PTCs such as papillary microcarcinomas [12,13,72,73,74,75,76]. (2) *BRAFV600E* mutation was identified in a large proportion (30–90%) of all PTCs, which resulted in poor outcomes in less than 10% of cases. (3) Only 29.8% of 47 PTCs with distant metastasis had *BRAF* mutation, which was less than that (44%) of control PTCs without distant metastasis reported by Sancis et al. [77]. (4) *BRAF* mutation is often an oligoclonal event in PTCs and metastases do not always maintain the mutation [70,78]. (5) *BRAF* mutation was absent in some fatal papillary microcarcinomas reported by Piana et al. [79]. However, most studies from Western thyroid practice claimed a significant association between the *BRAFV600E* mutation and poor outcome of PTC [4,80,81,82,83,84,85], even though this association was not reproducible in most Asian thyroid practices [7,8,9,10,11,12,13] and in some Western patient cohorts [14,86,87,88,89]. The author believes this discrepant observation was, at least in part, due to the marked observer variation in biologically indolent follicular variant PTC in the *BRAF* wild type group, where cases with delicate nuclear features of *RAS*-like tumors were classified as follicular variant PTCs.

The editor emphasizes the diagnostic value of *BRAF* gene testing in the preoperative diagnosis of classic/conventional PTCs using fine-needle aspiration specimens [90,91,92,93]. However, the implementation of *BRAF* mutation as a poor prognostic marker will increase risks to the patient as many low-risk PTCs have this mutation. In a recent opinion paper by Brazilian experts on papillary thyroid microcarcinoma, they stated that molecular tests are not necessary to select between active surveillance and surgery or, in the latter case, between lobectomy and total thyroidectomy. They further recommend that, in cases in which molecular tests are obtained, the presence of *RAS* or other *RAS*-like mutations or *BRAFV600E* or other *BRAF V600E*-like mutations should not affect management [94]. Please note that the 2015 American Thyroid Association clinical guidelines revised the treatment modality from total thyroidectomy to lobectomy for low-risk PTCs (including <4-cm intrathyroidal PTC and PTC with <5 nodal micro-metastasis) and the *BRAF* mutation was not a major determinant to identify PTCs of intermediate risk or high risk for structural disease recurrence [95].

## 7. Perspectives

There are significant differences in thyroid nodule practice among countries. However, the standardization of thyroid FNA cytology and histopathological diagnostic systems, without knowing the different social background, among countries maybe not be the best solution. The author of this commentary hopes all pathologists and cytopathologists understand the diversity in thyroid nodule practice and select reporting systems and diagnostic criteria that best suit the patient, which is the only method to provide tailored clinical management [96].

The author of this commentary proposes that all prognostic analyses of thyroid carcinomas before 2017 be re-evaluated because most prognostic data in the current clinical guidelines were established in Western patient cohorts where a significant number of indolent *RAS*-like tumors (NIFTP, UMP, minimally invasive FTC and minimally invasive encapsulated follicular variant PTC) contaminated the malignant category.

## Figures and Tables

**Table 1:** Incidence of *BRAFV600E* mutation in PTC variants.

Author [Reference Number]	Country	Classic	Tall Cell	Columnar Cell	Follicular
Xia [65]	USA		78%		
Fernandez [66]	Italy	77.4%	72.2%		31.9%
Villar-Taibo [67]	Spain		80%		28%
Virk [68]	USA	75%	91%	70%	21%
Adeniran [4]	USA		100%		
Jin [69]	Korea		92%	72.7%	
